# Transcript Abundance of *IL6*, *CXCL9*, *LIF* and *LIFR* in Glottic Lesions: Relationship to Histology and to Narrow-Band Imaging Vascular Patterns

**DOI:** 10.3390/cancers18213545

**Published:** 2026-11-04

**Authors:** Magda Barańska, Edyta Borkowska, Wioletta Pietruszewska

**Affiliations:** 1 Department of Otolaryngology, Head and Neck Oncology, Medical University of Lodz, 90-153 Lodz, Poland; wioletta.pietruszewska@umed.lodz.pl; 2 Department of Clinical Genetics, Medical University of Lodz, 92-213 Lodz, Poland; edyta.borkowska@umed.lodz.pl

**Keywords:** laryngeal squamous cell carcinoma, *IL-6*, *CXCL9*, *LIF/LIFR*, narrow band imaging, RT-qPCR

## Abstract

Background: Narrow-band imaging (NBI) is an established adjunct in laryngeal lesion assessment, but the molecular processes underlying high-risk vascular patterns are poorly defined. We compared *IL6*, *CXCL9*, *LIF*, and *LIFR* transcript abundance between glottic carcinoma and non-cancerous lesions, related it to NBI patterns, and tested whether *IL6* adds diagnostic information to NBI. Methods: In this prospective single-center exploratory study, 62 patients operated on for glottic lesions (33 squamous cell carcinomas, 29 non-cancerous lesions) underwent preoperative NBI (European Laryngological Society and Ni classifications); transcripts were quantified by RT-qPCR. Results: *IL6* was higher in carcinoma (median 5.97 vs. 0.30; *p* = 0.004), also with non-detects set to the detection limit (*p* = 0.001) and among ever-smokers only (*p* = 0.0008). Sex adjustment attenuated the association (OR 1.78 per 10-fold increase, 95% CI 0.98–3.23; *p* = 0.06); smoking could not be adjusted for, as all carcinomas occurred in ever-smokers. *CXCL9*, *LIF*, and *LIFR* did not differ. Adding *IL6* to NBI did not improve discrimination (ΔAUC 0.00–0.02; bootstrap 95% CIs including zero). Within non-cancerous lesions, *IL6* was nominally higher in high-risk NBI patterns (n = 5–7), but no stratified comparison survived false-discovery-rate correction. Conclusions: *IL6* transcript abundance was higher in glottic carcinoma, but the difference was sensitive to sex adjustment and, when measured in bulk tissue, may reflect lesion composition, including inflammatory infiltrate. *IL6* showed no evidence of diagnostic value beyond NBI, and its link with vascular morphology remains a hypothesis.

## Simple Summary

Cancer of the larynx is often preceded by vocal fold changes that look alike during examination. Narrow-band imaging is an endoscopic technique that highlights small mucosal blood vessels and helps identify lesions likely to be malignant, but it is unclear which biological processes these vessel patterns reflect. We measured the expression activity of four genes involved in inflammation and blood-vessel formation in tissue from 62 patients operated on for vocal fold lesions. The *IL6* gene expression was higher in cancer than in non-cancerous lesions, although this may partly reflect inflammatory cells within the tissue and the higher proportion of men and smokers among cancer patients. Adding *IL6* measurement to the endoscopic assessment did not improve cancer detection. A possible link between *IL6* and high-risk vessel patterns was seen in only a few lesions and needs confirmation in a larger study.

## 1. Introduction

Laryngeal squamous cell carcinoma (LSCC), the most common malignant tumor of the larynx, remains a substantial cause of morbidity and mortality, with outcomes strongly dependent on stage at diagnosis. According to GLOBOCAN 2022 estimates, cancer of the larynx accounted for approximately 189,000 new cases and 103,000 deaths worldwide, with age-standardized incidence and mortality rates of 1.9 and 1.0 per 100,000 person-years [1]. This supports continued investigation of biomarkers that may complement endoscopic and histopathological assessment, particularly in clinically heterogeneous premalignant and early malignant glottic lesions.

The transition from chronic mucosal inflammation through dysplasia to invasive carcinoma is accompanied by progressive remodeling of the epithelial and stromal microenvironment, including the subepithelial vasculature [2,3]. Narrow-band imaging (NBI) exploits the differential absorption of approximately 415 nm and 540 nm light by hemoglobin to enhance mucosal vascular contrast and is an established adjunct to white-light laryngoscopy in laryngeal lesion assessment [4,5,6]. Two commonly used descriptive systems are the European Laryngological Society (ELS) classification, which distinguishes longitudinal from perpendicular vascular changes, and the Ni classification, in which increasingly irregular intra-epithelial papillary capillary loops are associated with dysplastic or malignant transformation [6,7,8].

Recent single-cell RNA sequencing and spatial transcriptomic analyses of vocal cord polyps, leukoplakia and LSCC have demonstrated substantial epithelial, immune and stromal heterogeneity across benign, preneoplastic and malignant states, reinforcing the need to interpret bulk-tissue cytokine expression in relation to cellular composition [9]. More recent spatial and single-cell work in smoking-associated LSCC has also identified direct tumor–vascular crosstalk, further supporting the biological relevance of linking molecular signals with vascular phenotypes [10].

Although the diagnostic performance of NBI in laryngeal neoplasia is well characterized, the biological processes associated with the visible vascular phenotype remain incompletely defined [6,11]. IL-6 signaling through IL-6R/gp130 activates JAK/STAT3, MAPK and PI3K/AKT pathways and is implicated in tumor-cell survival, immune regulation and angiogenesis [12,13,14,15,16]. LIF and LIFR also signal through gp130; their effects are context-dependent, and recent HNSCC work supports a LIF/LIFR–STAT3–SOX2 regulatory loop rather than a uniformly tumor-suppressive or tumor-promoting role [17,18]. CXCL9 is an IFN-γ-induced CXCR3 ligand involved in cytotoxic lymphocyte recruitment, but its prognostic and functional effects vary according to tumor type, cellular source and immune context [19,20].

The novelty of the present study lies in four linked features. First, it simultaneously quantifies *IL6*, *CXCL9*, *LIF* and *LIFR* transcripts in clinically encountered glottic lesions, with benign, inflammatory and dysplastic lesions as the comparator. Second, it relates transcript abundance to vascular morphology using both the ELS and Ni systems, including a stricter Ni type 5 threshold. Third, it evaluates whether *IL6* adds diagnostic information beyond NBI rather than assuming that a molecular association implies clinical utility. Fourth, it provides an RNA-level perspective that is analytically distinct from our own recently published immunohistochemical study of the same cytokine panel in 145 vocal-fold lesions, in which Ni ≥ 4 was the strongest independent predictor of SCC while epithelial IL-6 showed near-chance discrimination and *LIF/LIFR* showed inverse associations with malignancy [21]. The primary aim was to compare transcript abundance between carcinoma and non-cancerous lesions; secondary exploratory aims were to assess associations with NBI patterns, smoking exposure and inter-gene co-expression, and to quantify the incremental diagnostic contribution of *IL6*.

## 2. Materials and Methods

### 2.1. Study Design and Patient Enrolment

This was a prospective single-center observational study conducted at the Department of Otolaryngology, Head and Neck Oncology, Medical University of Lodz, Poland. Consecutive patients qualified for surgical management of vocal fold lesions between November 2020 and June 2023 were screened for eligibility. Inclusion criteria were: (i) age ≥ 18 years; (ii) clinical indication for surgical biopsy or excision of a vocal fold lesion; and (iii) availability of a high-quality preoperative endoscopic examination in both white-light and NBI modes. Exclusion criteria were: (i) prior diagnosis of head and neck malignancy; (ii) previous radiotherapy or chemotherapy of the head and neck; (iii) active infectious disease at the laryngeal level; (iv) systemic immunosuppression; (v) endoscopic recordings of insufficient quality for vascular pattern scoring; and (vi) refusal of informed consent. Sample size was determined by patient availability within the recruitment window; no formal a priori power calculation was performed, and the analysis is therefore explicitly framed as exploratory.

With 33 carcinoma and 29 non-cancerous lesions, the smallest standardized difference detectable at 80% power and a two-sided α of 0.05 is approximately 0.73 SD. The histology-stratified analyses rest on five to seven lesions per stratum and are reported as exploratory throughout. The four-marker panel and the two NBI classification systems used here were also examined at protein level by our own group in a separate immunohistochemical study of 145 vocal fold lesions from the same department [21]. The two series partly overlap: 21 of the 62 patients reported here also contributed material to that study, and 41 are unique to the present cohort, the 21 shared patients representing 14% of the 145 lesions reported in [21]; the present cohort is therefore neither a subset of, nor fully independent of, the earlier series. Both studies recruited at the same department over the same period (November 2020 to June 2023), but material for each was collected separately and could be secured only when a member of the study team was available at surgery. For RT-qPCR, an additional fresh fragment had to be stabilized in RNAlater intraoperatively without compromising the specimen submitted for diagnosis, which in transoral laser microsurgery of small lesions excised with narrow margins is frequently not possible. Inclusion in either series therefore depended on the logistics of intraoperative sampling, which explains the limited overlap between the two cohorts. The two studies share authorship.

Histologically normal vocal fold mucosa was not included as a comparator. Vocal fold epithelium and Reinke’s space cannot be sampled in healthy volunteers without risk of permanent voice impairment, and mucosa adjacent to a carcinoma would be subject to field-cancerization effects. The study therefore compared carcinoma with benign, inflammatory and dysplastic lesions of the kind that NBI is used to assess, and not with healthy mucosa. Because 19 of 33 carcinomas were T3–T4 and 15 of 29 non-cancerous lesions were Reinke’s oedema or polyps, the comparison is weighted toward clinically evident cases. The comparator is also biologically conservative, since it contains inflammatory lesions in which *IL6* may be expressed.

The study was performed in accordance with the Declaration of Helsinki and approved by the Bioethics Committee of the Medical University of Łódź (decision RNN/257/20/KE). All participants provided written informed consent.

### 2.2. Endoscopic Assessment and NBI Scoring

Transnasal flexible video-endoscopy was performed on the day of surgery using an ENF-VH2 endoscope (Olympus Corporation, Tokyo, Japan) connected to a video processor with NBI capability. Examinations were carried out by an experienced laryngologist; topical lidocaine spray was administered when required to suppress the gag reflex. White-light evaluation preceded NBI activation, and still images of every lesion were archived for later scoring.

Vascular morphology was scored by three observers blinded to histopathological results, who reviewed the archived images together and agreed on a single classification for each lesion by consensus. The European Laryngological Society descriptive guideline was applied as a binary system distinguishing longitudinal vessels (parallel to the epithelial surface, considered low-risk) from perpendicular vessels (dot-like, spiral or corkscrew patterns, considered high-risk) [7]. The Ni classification was applied as a five-level ordinal scale; for analysis, lesions were grouped into low-grade (types 1–3) and high-grade (types 4–5) categories [8]. The individual observers’ classifications were not entered into the study database, so quantitative interobserver agreement cannot be reconstructed retrospectively; this is acknowledged as a limitation.

### 2.3. Tissue Sampling and Surgical Procedures

Surgical procedures were planned according to lesion size, sub-site and clinical risk: transoral laser microsurgery (TOLMS) cordectomies were performed under general anesthesia and classified following the ELS system into types I–VI; partial laryngectomies and total laryngectomies were performed where indicated. Representative tissue specimens of approximately 3–5 mm^3^, depending on the size of the lesion, were obtained from the central, non-necrotic and non-hemorrhagic portion of each lesion. Specimens were placed immediately in RNAlater Stabilization Solution (Ambion, Thermo Fisher Scientific, Waltham, MA, USA), kept at 4 °C overnight, and stored at −20 °C until RNA extraction. All histopathological diagnoses were issued by a board-certified pathologist using hematoxylin–eosin sections and supplementary stains where appropriate and reported according to the WHO Classification of Head and Neck Tumours, 5th edition [22].

### 2.4. RNA Isolation, Reverse Transcription and Quantitative PCR

Total RNA was extracted from tissue fragments using the Maxwell RSC simplyRNA Tissue Kit on the Maxwell RSC Instrument (Promega Corporation, Madison, WI, USA), following the manufacturer’s protocol; all manipulations were performed under RNase-free conditions. Genomic DNA was removed by DNase I treatment as part of the Maxwell RSC simplyRNA Tissue protocol, and RNA concentration was measured after this step. RNA concentration and purity were verified spectrophotometrically with a NanoDrop 2000 instrument (Thermo Fisher Scientific, Waltham, MA, USA); samples with A260/A280 between 1.8 and 2.1 and A260/A230 ≥ 2.0 were accepted for downstream analysis. RNA integrity was assessed using an electrophoretic RNA integrity number (RIN) metric. Samples with RIN ≥ 6.0 were accepted for analysis; observed values ranged from 6.0 to 7.0.

Reverse transcription was carried out using the GoTaq 2-Step RT-qPCR System (Promega, Madison, WI, USA), following the manufacturer’s instructions; the volume of RNA was adjusted individually to obtain 100 ng of total RNA per reaction. cDNA was used at a 1:5 dilution in the qPCR reaction mix. Quantitative real-time PCR was performed on a Bio-Rad CFX96 Touch Real-Time PCR Detection System (Bio-Rad Laboratories, Hercules, CA, USA) using TaqMan Fast Advanced Master Mix and pre-designed TaqMan Gene Expression Assays (Thermo Fisher Scientific): *IL6* (Hs00174131_m1), *CXCL9* (Hs00171065_m1), *LIF* (Hs01055668_m1), *LIFR* (Hs01123581_m1) and the endogenous reference *GAPDH* (Hs02786624_g1). Cycling conditions were: 95 °C for 20 s (initial denaturation) followed by 40 cycles of 95 °C for 1 s and 60 °C for 20 s (combined annealing and extension). All reactions were performed in technical duplicate with no-template controls included on every plate. Quantification cycle (Cq) values were determined automatically using Bio-Rad CFX Maestro software (version 2.1). 

Relative expression was calculated by the 2^−ΔΔCt^ method with *GAPDH* as the reference gene. The calibrator was not a single pooled specimen but the mean ΔCq of a separate reference set, computed independently for each target; the reference set comprised specimens of healthy laryngeal mucosa obtained from distant resection margins, together with control specimens, none of which was among the 62 lesions analyzed here. The same reference value was applied to every plate for a given target. Because expression values are referenced in this way, both the reported 2^−ΔΔCt^ values and the *IL6* threshold derived below are specific to this reference set and cannot be applied in another laboratory without re-derivation against it. Since margins from laryngectomy specimens come from cancer-affected laryngeal tissues, they may be affected by field cancerization. Current MIQE guidance requires transparent validation and reporting of reference-gene selection, assay performance, and reaction-quality criteria [23,24]. Amplification efficiency was monitored as part of assay quality control, and only assays with efficiencies within the 90–110% range were accepted for analysis. Endpoint RT-PCR gel images were not generated, as quantification was based on hydrolysis-probe real-time PCR.

### 2.5. Statistical Analysis

Descriptive statistics, Mann–Whitney comparisons, correlation analyses, and receiver operating characteristic curves were performed in GraphPad Prism 10 (GraphPad Software, Boston, MA, USA), with selected results verified in Statistica 13.1 (StatSoft, Kraków, Poland). Logistic regression models, nested likelihood-ratio tests, multiplicity correction, bootstrap resampling, and the sensitivity analyses reported here were performed in Python (version 3.14) using the stats models, SciPy, scikit-learn, and pandas libraries; figures were prepared in Python with matplotlib and seaborn. Continuous variables are reported as median with IQR. Categorical variables are reported as counts and percentages. The primary comparison (cancer vs. non-cancerous) used the Mann–Whitney U test, with effect size reported as the rank-biserial correlation coefficient. Correlations between genes were assessed by Spearman’s ρ. To address sex confounding, binary logistic regression was performed with log10-transformed expression values as the predictor; models were run with and without sex as a covariate, and results are reported as odds ratios (ORs) with 95% Wald confidence intervals. The logistic models included the specimens with detectable *IL6* (n = 57; 32 carcinomas and 25 non-cancerous lesions); non-detects were excluded. Because sex and smoking status were markedly imbalanced between histological groups, these models were interpreted as exploratory rather than as proof of an independent association. A *p*-value < 0.05 was considered significant. The histology-stratified comparisons were corrected for multiple testing using the Benjamini–Hochberg procedure across all estimable tests, and q-values are reported alongside nominal *p*-values. An additional sensitivity analysis was performed for the handling of non-detects (assignment to the limit of detection). The comparison of *IL6* between histological groups was repeated in ever-smokers only. Diagnostic performance was recomputed on the subset evaluable for all classifiers, with Wilson 95% confidence intervals; ΔAUC and the optimism of the *IL6* AUC were estimated by bootstrap with 2000 resamples. The difference between the *LIF/LIFR* correlation coefficients of the two groups was tested by Fisher r-to-z transformation. The primary *IL6* cancer-vs-non-cancer comparison survived Bonferroni correction for four genes (adjusted α = 0.0125, primary *p* = 0.004).

## 3. Results

### 3.1. Cohort Characteristics

Sixty-two patients fulfilling inclusion criteria were enrolled between November 2020 and June 2023; all lesions were located at, or originated from, the glottis. Demographic and clinico-pathological features are summarized in Table 1. The cohort comprised 42 men and 20 women, with a median age of 68 years (interquartile range, IQR, 59–73).

Final histopathology classified 33 patients as squamous cell carcinoma (SCC) and 29 as non-cancerous lesions. For the primary analysis, the cohort was dichotomized into cancer (n = 33) and non-cancerous (n = 29) groups by final histopathological diagnosis. NBI category was strongly associated with histological diagnosis: ELS-perpendicular vessels predominated in cancer (32/33), whereas longitudinal vessels predominated in non-cancerous lesions (20/29); Ni types 4–5 predominated in cancer (28/33) and Ni types 1–3 in non-cancerous lesions (23/29). Using the stricter Ni threshold, Ni type 5 was observed in 26/33 carcinomas and in only 2/29 non-cancerous lesions, whereas Ni types 1–4 were observed in 7/33 carcinomas and 27/29 non-cancerous lesions.

### 3.2. IL6 Transcript Abundance Differs Between Cancerous and Non-Cancerous Lesions

In the primary comparison (Figure 1), *IL6* expression was higher in carcinoma than in non-cancerous tissue (median 5.97, IQR 0.97–24.95 vs. 0.30, IQR 0.07–6.26; Mann–Whitney *p* = 0.004; rank-biserial correlation = 0.45, with positive values denoting higher expression in carcinoma). *CXCL9* (6.06 vs. 5.47; *p* = 0.71), *LIF* (1.65 vs. 2.15; *p* = 0.64) and *LIFR* (0.20 vs. 0.60; *p* = 0.15) did not differ significantly. The *IL6* result remained below the Bonferroni-adjusted threshold for four primary gene comparisons (α = 0.0125). In logistic regression, each 10-fold increase in *IL6* was associated with higher crude odds of carcinoma (OR 2.06, 95% CI 1.22–3.48; *p* = 0.007); after adjustment for sex, the estimate was attenuated (OR 1.78, 95% CI 0.98–3.23; *p* = 0.06). Given the small sample, strong sex imbalance and separation of smoking status by diagnosis, the adjusted model should be interpreted cautiously. Restricting the comparison to ever-smokers, which removes the never-smoker stratum present only among non-cancerous lesions, gave a larger rather than a smaller separation (median *IL6* 5.97, n = 32, versus 0.23, n = 19; *p* = 0.0008). The carcinoma with a longitudinal ELS pattern, which showed extreme values for all four transcripts, was retained in all analyses.

The number of evaluable specimens differed between transcripts (Table 2). A non-detect identifies a specimen with expression below the detection limit for that transcript, so excluding such specimens is informative censoring, and the proportion censored differed between groups (for example, six of 29 non-cancerous specimens versus none of 33 carcinomas for *CXCL9*). We therefore repeated the primary comparisons with non-detects assigned the limit of detection. The *IL6* difference strengthened (*p* = 0.001, rank-biserial correlation 0.48, n = 33 versus 29, compared with *p* = 0.004 and 0.45 in the complete-case analysis). *CXCL9* moved toward, but did not reach, significance (*p* = 0.09 versus *p* = 0.71), consistent with the censoring having acted asymmetrically for that transcript; *LIF* (*p* = 0.48) and *LIFR* (*p* = 0.18) were essentially unchanged. Because the comparisons are rank-based, these results do not depend on the value assigned to the non-detects, provided it lies below all observed values. Raw *GAPDH* Cq was lower, that is, reference-gene abundance was higher, in carcinoma than in non-cancerous lesions (median 27.6, IQR 25.5–31.2 versus 30.4, 28.5–32.5; *p* = 0.05).

### 3.3. NBI Stratified by Histological Group

To examine whether transcript–NBI associations were observable within histological strata, NBI comparisons were repeated separately in the carcinoma and non-cancerous groups (Table 3). Within carcinoma, the ELS category was almost invariant (only one longitudinal case), precluding a meaningful ELS comparison; Ni comparisons were underpowered and non-significant. Within the non-cancerous group, *IL6* was higher in ELS-perpendicular lesions (n = 7) and in Ni 4–5 lesions (n = 5), and *CXCL9* was higher in ELS-perpendicular lesions. Across the 20 estimable stratified tests, however, no result survived Benjamini–Hochberg correction; the smallest q-value was 0.27. These subgroup analyses rest on small numbers and, although corrected for multiple testing, should be treated as exploratory.

Because vascular pattern and dysplasia might coincide within the non-cancerous group, the stratified comparisons were repeated with dysplasia status taken into account. Of the nine non-cancerous lesions with perpendicular vessels, seven were dysplastic (six high-grade, one low-grade), and of the six with Ni 4–5, five were dysplastic. *IL6* abundance did not, however, differ by dysplasia status (median 0.43 in dysplastic versus 0.30 in non-dysplastic non-cancerous lesions; *p* = 0.80), and the direction of the vascular contrast was preserved within both strata (dysplastic lesions: 10.79, n = 5, versus 0.07, n = 3; non-dysplastic lesions: 47.89, n = 2, versus 0.23, n = 15).

### 3.4. Diagnostic Performance of NBI and IL6

With carcinoma as the positive class, NBI discriminated well under both Ni thresholds (Table 4). On the subset evaluable for all four classifiers (n = 57; 32 carcinoma, 25 non-cancerous), the ELS classification was the most sensitive (96.9%, 95% CI 84.3–99.4) and the Ni 5 vs. 1–4 threshold the most specific (92.0%, 95% CI 75.0–97.8). Ni 5 vs. 1–4 gave the highest Youden index (0.73), with ELS (0.69) and Ni 4–5 vs. 1–3 (0.68) essentially equivalent. Thus, Ni 4–5 vs. 1–3 represents the clinically broader high-risk threshold, whereas Ni 5 vs. 1–4 is a stricter threshold with higher specificity but lower sensitivity. *IL6* on its own separated carcinoma from non-cancerous tissue with an AUC of 0.725 (95% CI 0.57–0.86); at the Youden-optimal cut-off (≥0.52), its apparent sensitivity and specificity were 93.8% and 56.0%; sensitivity was below that of ELS and specificity below all three NBI classifiers (Figure 2A).

Adding *IL6* to NBI did not significantly improve diagnostic discrimination. Bootstrap confidence intervals for the change in AUC on adding *IL6* all included zero: ELS +0.004 (95% CI −0.029 to +0.098), Ni 5 vs. 1–4 +0.022 (−0.009 to +0.082), Ni 4–5 vs. 1–3 +0.000 (−0.027 to +0.083) and the ordinal Ni model +0.003 (−0.021 to +0.052), with likelihood-ratio *p*-values of 0.44, 0.36, 0.50 and 0.63 respectively. The upper bounds of these intervals reach +0.08 to +0.10, so this cohort provides no evidence of incremental diagnostic value beyond NBI but is not large enough to exclude a contribution of that size. Using the ordinal Ni score, NBI alone and NBI plus *IL6* both yielded an AUC of 0.90, compared with 0.72 for IL6 alone (Figure 2B). Because these estimates were derived and evaluated in the same small cohort, they are interpreted as exploratory and require validation in an independent dataset. Optimism correction by bootstrap changed the *IL6* AUC only marginally (0.724 corrected versus 0.725 apparent).

*IL6* did not distinguish non-cancerous lesions with a perpendicular vascular pattern (n = 7) from carcinomas sharing that pattern (n = 31); the difference was, in fact, numerically in the opposite direction, with higher *IL6* in the non-cancerous lesions (median 10.79 versus 5.49; *p* = 0.88). *IL6* was higher in non-cancerous lesions with perpendicular versus longitudinal vessels and with Ni 4–5 versus Ni 1–3 patterns, whereas no Ni-related difference was detected within carcinoma. This pattern is compatible with an association between *IL6* abundance and vascular morphology, but it does not establish that *IL6* reflects angiogenesis rather than dysplasia, inflammation or tissue composition. Overall, this cohort provided no evidence that *IL6* improves carcinoma discrimination beyond NBI.

### 3.5. Smoking Exposure Stratified by Histological Group

Cancer patients had greater cumulative smoking exposure than non-cancer patients (median 30 vs. 20 pack-years; *p* = 0.010), and all carcinomas occurred in current or former smokers, whereas all seven lifelong non-smokers were in the non-cancer group (Figure 3). No statistically significant within-group correlation between pack-years and *IL6* was detected (carcinoma ρ = 0.29, *p* = 0.11; non-cancer ρ = 0.04, *p* = 0.84). *IL6* abundance in the six evaluable never-smokers, all in the non-cancerous group, was high but variable (median 38.41; Table 5); this group is too small for further analysis. These null within-group results do not exclude confounding because the subgroups were small and smoking status was strongly imbalanced by diagnosis. The inverse association between *LIF* and pack-years in the non-cancer group (ρ = −0.56; *p* = 0.003) arose from secondary multiple testing and should be considered exploratory.

Within histological groups, neither *IL6* nor NBI category showed a statistically significant association with cumulative smoking exposure (Table 5, Figure 4). These analyses reduce, but do not eliminate, concern about smoking-related confounding because all carcinomas occurred in ever-smokers and the study had limited power for within-group associations. The *LIF*–smoking association in non-cancerous lesions should be interpreted cautiously and requires independent replication.

### 3.6. LIF and LIFR Show the Strongest Within-Group Co-Expression

Spearman correlations were calculated separately in carcinoma and non-cancerous lesions to avoid correlations induced by pooling groups with different expression distributions. *LIF* and *LIFR* showed the strongest correlation in both groups (ρ = 0.73 and 0.86; both *p* < 0.001; Figure 5). The two coefficients did not differ significantly between groups (Fisher r-to-z: z = −1.25, *p* = 0.21; n = 28 and 23 evaluable pairs). Other nominally significant correlations were less consistent and were not corrected for multiple testing. The *LIF/LIFR* finding is biologically plausible, but shared normalization to a single reference gene can induce positive correlations among normalized targets. The observation should therefore be validated using multiple stable reference genes, raw or efficiency-corrected Cq-based analyses, and protein-level measurements.

## 4. Discussion

This prospective study measured transcript abundance of four cytokine-pathway genes in surgically managed glottic lesions and related it to two NBI vascular classification systems. *IL6* was higher in carcinoma than in non-cancerous lesions, but adding it to NBI did not improve discrimination, and its association with vascular morphology within histological strata was not robust. The strengths of the study are a comparator group of non-dysplastic, inflammatory and dysplastic lesions from a surgical series, the parallel use of the ELS and Ni systems, and a formal test of diagnostic increment over NBI.

### 4.1. IL6 and High-Risk Vascular Morphology

The higher *IL6* transcript abundance in carcinoma is consistent with previous evidence implicating IL-6 signaling in HNSCC and laryngeal cancer [14,25,26]. IL-6 can activate JAK/STAT3-related programs linked to survival, inflammation and angiogenesis, and experimental work has shown that IL-6 can directly promote aberrant angiogenic responses [13]. Direct laryngeal-cancer evidence has expanded further: a 2025 functional study identified a circHIPK2/miR-889-3p/MCTS1 axis that increased IL-6 secretion and promoted LSCC-cell proliferation and migration [27]. In parallel, spatial and single-cell transcriptomic analysis of smoking-associated LSCC has demonstrated tumor–vascular signaling linked to JAK/STAT activity [10]. These data strengthen the biological plausibility of an *IL6*–vascular association, but they do not establish that tissue *IL6* transcript abundance causes the NBI vascular patterns observed in the present cross-sectional cohort. The present study did not measure IL-6 protein, STAT3 or HIF1 activation, VEGF, endothelial markers, microvessel density, or the cellular source of IL6 [28,29,30].

Because NBI category and histological diagnosis were strongly collinear, the pooled *IL6*–NBI association carries no information beyond the carcinoma versus non-carcinoma contrast, and the only evidence bearing on an NBI-specific association is the histology-stratified analysis. The two supporting results rest on seven ELS-perpendicular and five Ni 4–5 non-cancerous lesions; across the 20 estimable stratified tests, none survives Benjamini–Hochberg correction, the smallest q being 0.27. In addition, *IL6* non-detects were more frequent among non-cancerous lesions with high-risk patterns (2 of 9 ELS-perpendicular and 1 of 6 Ni 4–5 lesions) than among those with low-risk patterns (2 of 20 and 3 of 23), so the complete-case stratified comparisons removed the lowest values mainly from the high-risk groups and may overestimate the association. The association is absent at the stricter Ni 5 threshold, where an association tracking vascular severity would be expected to be strongest. Two contrasts also run in the opposite direction: within the perpendicular phenotype, *IL6* was numerically higher in non-cancerous lesions than in carcinomas (10.79 versus 5.49), and the single carcinoma with a low-risk longitudinal pattern had the highest *IL6* in its group, together with extreme values for three other transcripts, which points to a normalization artefact rather than biology.

Of the nine non-cancerous lesions with perpendicular vessels, seven were dysplastic, and of the six with Ni 4–5, five were; the vascular and histological strata therefore overlap substantially. However, *IL6* did not track dysplasia in this cohort (median 0.43 versus 0.30, *p* = 0.80), and the direction of the vascular contrast was preserved within both dysplasia strata. The within-stratum association therefore does not reduce simply to the histological contrast already reported, although with five, three, two and fifteen lesions in the four cells this is an observation rather than a demonstration.

Taken together, these data support a difference in *IL6* transcript abundance between glottic carcinoma and non-cancerous lesions. They do not establish *IL6* as a correlate of the NBI vascular phenotype, and that relationship should be regarded as a hypothesis generated by a small number of lesions and tested in an adequately powered series. Unlike many biomarker studies, the present analysis tested whether the marker adds to an established clinical tool. *IL6* did not improve discrimination beyond NBI in this cohort, suggesting that, at the level of carcinoma classification, the transcript signal and the optical phenotype carry largely overlapping information.

### 4.2. The Non-Discriminating Transcripts and the LIF–LIFR Correlation

*CXCL9*, *LIF* and *LIFR* did not differ significantly between carcinoma and non-cancerous lesions (Figure 1), while the histology-stratified NBI comparisons are summarized in Table 3. This result should not be interpreted as evidence that these pathways are unimportant. The comparator group contained dysplastic and inflammatory lesions rather than histologically normal mucosa, and bulk-tissue measurements combine epithelial, immune and stromal signals. Database-driven HNSCC studies reporting *CXCL9* upregulation or prognostic value are not specific to glottic carcinoma and often compare tumor with normal tissue; their findings therefore provide context but do not directly predict the present contrast [26,31]. Recent single-cell profiling of normal, preneoplastic and malignant laryngeal tissue further emphasizes that bulk-tissue expression represents a composite of epithelial, stromal and immune-cell states [9]. This strengthens the need for spatial or single-cell validation before assigning the observed *IL6*, *CXCL9*, *LIF* or *LIFR* signals to a specific cellular compartment.

Normalization to a single reference gene also has a direction in this setting. *GAPDH*, a glycolytic gene commonly upregulated in squamous carcinoma, was borderline more abundant in carcinoma than in non-cancerous lesions (Section 3.2), which biases all four normalized targets downward in the carcinoma group. The *IL6* difference is therefore unlikely to be a normalization artefact, whereas the negative results for *CXCL9*, *LIF* and *LIFR* may underestimate true differences. *GAPDH* Cq values were also high and spanned about six cycles within each group despite a fixed RNA input. This variability adds noise to all normalized values and may also explain the extreme values of the single carcinoma with a longitudinal pattern.

The discordance with our own protein-level findings [21], where epithelial IL-6 discriminated poorly and the marker hierarchy differed, has several possible explanations that we cannot separate here. The cohorts overlap only partly, so differences in composition may contribute. The analyte and the compartment differ: immunohistochemistry quantified protein within the epithelium, whereas RT-qPCR measured transcript abundance in bulk tissue containing epithelium, stroma, and inflammatory infiltrate. Lesion-level selection also differed, since RT-qPCR required a spare fresh fragment that could not be obtained from every operated lesion. Resolving this discordance would require spatially resolved measurement of both analytes in the same lesions.

*LIF* and *LIFR* co-expression (Figure 5) is consistent with coordinated pathway activity and with recent HNSCC evidence for a LIF/LIFR–STAT3–SOX2 feedback loop [17]. Nonetheless, the correlation may be inflated by common normalization to *GAPDH* and does not show receptor activation or pathway direction. Notably, our earlier immunohistochemical study reported decreasing LIF and LIFR immunohistochemical expression with increasing histopathological severity and higher NBI-risk categories, whereas the present transcript-level study primarily identifies co-expression rather than a robust between-group difference [21]. This difference reinforces the value of orthogonal RNA- and protein-level assessment and argues for paired transcript/protein validation in future work.

The comparator group consisted of benign, inflammatory, and dysplastic lesions rather than histologically normal mucosa, and both the ELS and the Ni classifications were analyzed, allowing a sensitive broad threshold to be compared with the more specific Ni type 5 threshold. *IL6* abundance differed between histological groups, but the diagnostic increment over NBI was formally tested and not demonstrated.

### 4.3. Methodological Considerations and Limitations

The study has several important limitations. The histological groups differed markedly in sex and smoking status: 31 of 33 carcinoma patients were men, all carcinomas occurred in ever-smokers, and most women were in the non-cancerous group. The sex-adjusted *IL6* association was attenuated to *p* = 0.06, and the small sample did not permit robust multivariable control for sex, age, smoking, and histological composition. The *IL6* difference was not examined separately in men or by sex within the non-cancerous group, so the role of sex in the *IL6* difference remains unclear. Absence of a significant within-group correlation with pack-years does not exclude residual confounding. The non-cancerous group was heterogeneous and combined non-dysplastic and dysplastic lesions; this may confound both gene-expression and NBI comparisons.

Gene expression was assessed in bulk tissue by RT-qPCR using a single reference gene (*GAPDH*). No reference-independent analysis of raw Cq values and no validation of additional reference genes were performed, so the transcript results depend on normalization to *GAPDH* and should be confirmed using several validated reference genes. The fragment used for RNA extraction was not examined histologically; in small dysplastic lesions and in Reinke’s oedema it may have consisted mainly of stroma, which is relevant to the possibility that the *IL6* difference reflects tissue composition. The *LIF/LIFR* co-expression result warrants cautious interpretation because both targets were normalized to the same reference gene. Protein-level and spatial validation were not performed; therefore, the present study establishes transcript-level associations but cannot determine the cellular source or functional direction of these signals. The cohort is single-center and surgically selected, and the 53% carcinoma prevalence is a property of a tertiary referral series, so the predictive values reported here are not transportable to a screening setting. NBI was scored by consensus, so the diagnostic performance should be read as an upper bound relative to routine single-observer practice. The requirement for a spare fresh fragment favored larger lesions and plausibly contributes to the absence of T2 tumors and the over-representation of bulky T3/T4 disease. Together with the large proportion of Reinke’s oedema and polyps among the non-cancerous lesions, this case mix favors discrimination by both NBI and *IL6* and may overestimate their performance in early glottic lesions, in which NBI is most often used to resolve diagnostic uncertainty. The *IL6* threshold is expressed in calibrator-referenced units and is laboratory-specific. The association with *IL6* is unadjusted for smoking, the dominant exposure in this disease. Follow-up for malignant transformation of the non-cancerous lesions was not collected systematically and could not be reconstructed; since transformation, rather than cross-sectional discrimination, is the clinically decisive endpoint for these lesions, this remains the most important gap in the present cohort.

## 5. Conclusions

In 62 patients operated on for glottic lesions, *IL6* transcript abundance was higher in squamous cell carcinoma than in non-cancerous lesions (median 5.97 versus 0.30; *p* = 0.004; rank-biserial correlation 0.45), and the difference strengthened when transcript-specific non-detects were assigned the limit of detection (*p* = 0.001) and persisted when the comparison was restricted to ever-smokers (*p* = 0.0008). The association was not robust to adjustment for sex (OR 1.78 per 10-fold increase, 95% CI 0.98–3.23, *p* = 0.06), and smoking could not be adjusted for because all carcinomas occurred in ever-smokers (quasi-complete separation). *CXCL9*, *LIF* and *LIFR* did not differ between groups, although normalization to *GAPDH* biases these comparisons conservatively. *IL6* alone discriminated carcinoma with an AUC of 0.725, against 0.84 to 0.90 for the NBI classifiers, and adding *IL6* to NBI changed the AUC by 0.00 to 0.02 with bootstrap confidence intervals that included zero; this cohort therefore provides no evidence of incremental diagnostic value beyond NBI, while being underpowered to exclude a contribution of up to approximately 0.10 in AUC. Because the measurements were made in bulk tissue, the difference may reflect the cellular composition of the lesion, including its inflammatory infiltrate, rather than a tumor-cell-intrinsic program. *LIF* and *LIFR* were strongly co-expressed in both groups (ρ = 0.73 and 0.86; Fisher r-to-z *p* = 0.21). The relationship between *IL6* and high-risk vascular morphology rested on five to seven lesions and did not survive correction for multiple testing; it is presented as hypothesis-generating and requires an adequately powered study with spatial or single-cell resolution.

## Figures and Tables

**Figure 1:**
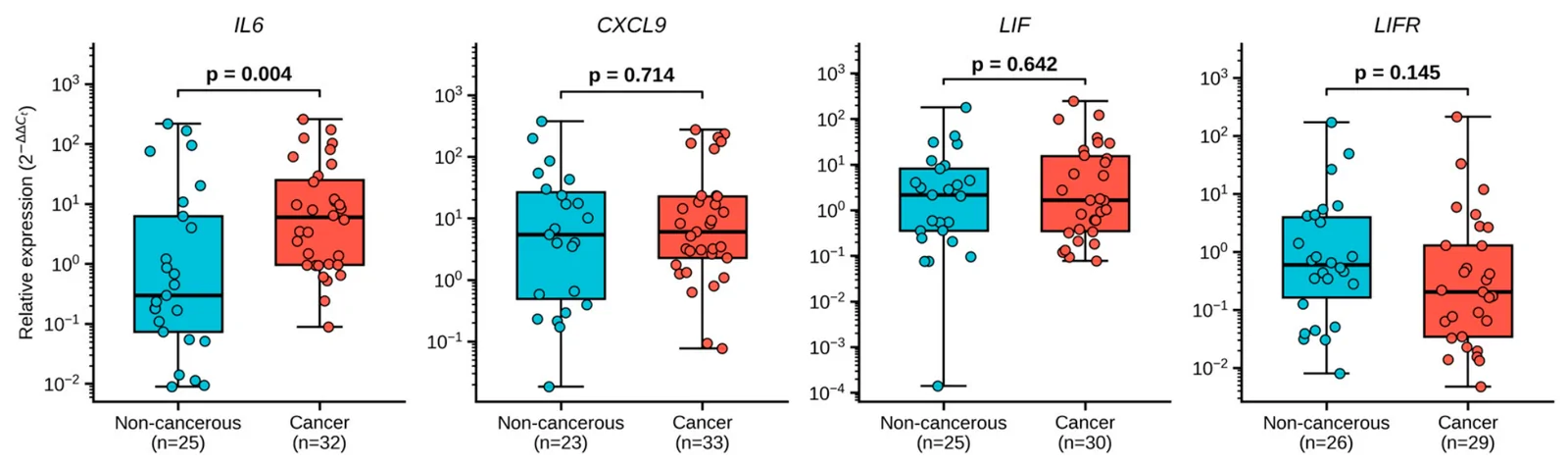
Expression of *IL6*, *CXCL9*, *LIF* and *LIFR* in cancerous (n = 29–33) and non-cancerous (n = 23–26) glottic lesions. Boxes show median and interquartile range; whiskers, 1.5 × IQR; points, individual samples (jittered). The y-axis is on a log10 scale. *p*-values are from Mann–Whitney U tests. Sample numbers vary between transcripts because only evaluable qPCR results were included in each comparison.

**Figure 2:**
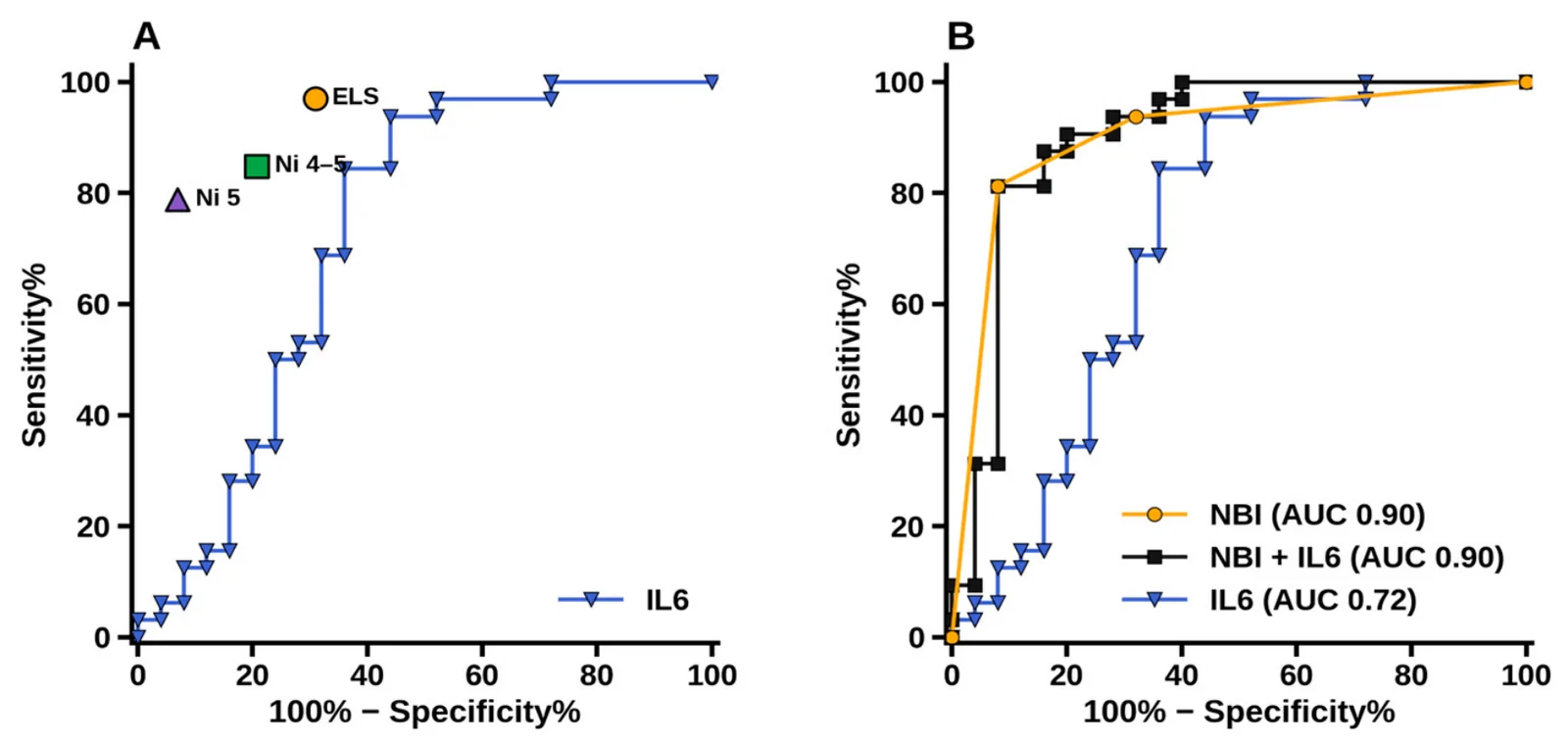
Diagnostic discrimination of carcinoma. (**A**) Receiver operating characteristic curve for *IL6* (n = 57), with the operating points of the three binary NBI classifiers superimposed: ELS perpendicular (circle), Ni 4–5 versus 1–3 (square) and Ni 5 versus 1–4 (triangle). The Youden-optimal *IL6* cut-off (≥0.52 in calibrator-referenced 2^−ΔΔCt^ units) corresponds to a sensitivity of 93.8% and a specificity of 56.0%. (**B**) Receiver operating characteristic curves (n = 57). “NBI” denotes a logistic model treating the Ni classification as an ordinal score (AUC 0.900), not any of the binary thresholds shown in panel (**A**). Adding log-transformed *IL6* abundance to that model did not improve discrimination (AUC 0.902; ΔAUC +0.003, 95% CI −0.021 to +0.052; likelihood-ratio *p* = 0.625). *IL6* alone gave an AUC of 0.725.

**Figure 3:**
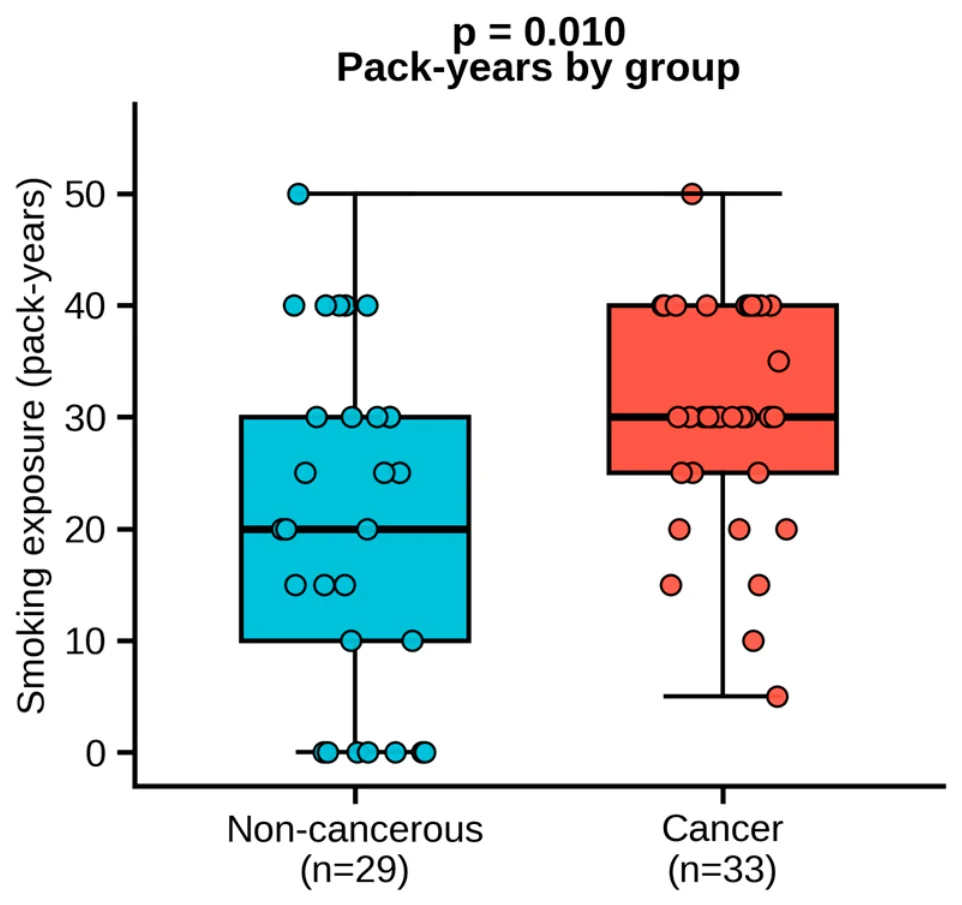
Cumulative smoking exposure by histological group. Pack-years were higher in patients with carcinoma than in those with non-cancerous lesions (median 30 versus 20; *p* = 0.010, Mann–Whitney U test). All 33 carcinomas occurred in ever-smokers, whereas all seven lifelong non-smokers were in the non-cancerous group.

**Figure 4:**
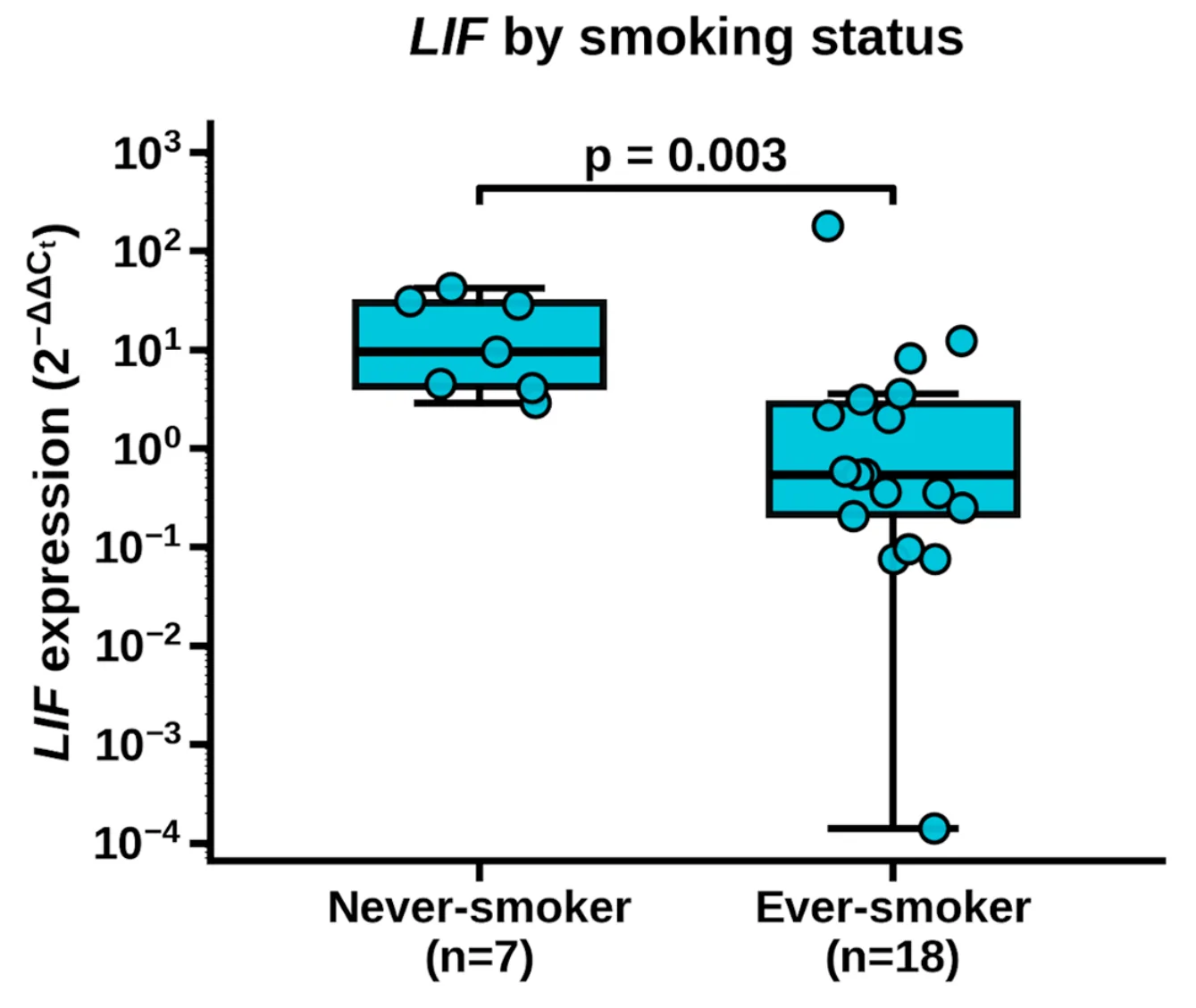
*LIF* expression in never-smokers and ever-smokers within the non-cancerous group (Mann–Whitney *p* = 0.003). Boxes show median and interquartile range; points are individual samples on a log10 scale.

**Figure 5:**
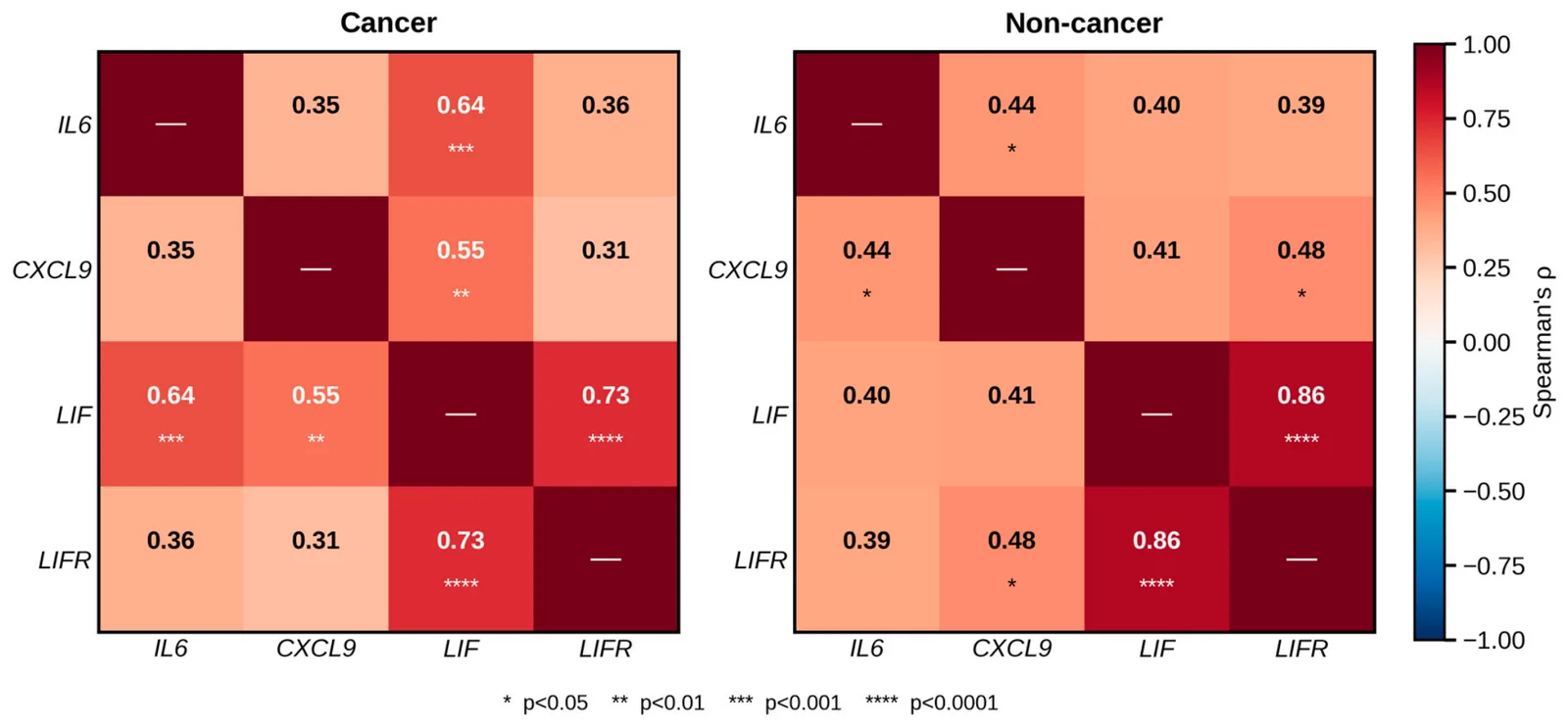
Spearman rank correlations between the four transcripts, computed separately for the cancer and non-cancer groups. Color intensity represents the strength of the correlation coefficient (ρ). The strongest and most consistent association was observed between *LIF* and *LIFR* in both groups. Significance levels: * *p* < 0.05, ** *p* < 0.01, *** *p* < 0.001, **** *p* < 0.0001. Correlations were computed pairwise on complete observations, so the number of specimens differs between cells: 28 to 32 in the carcinoma group and 20 to 23 in the non-cancerous group.

**Table 1:** Demographic and clinico-pathological characteristics of the study group.

Characteristic	Carcinoma (n = 33)	Non-Cancerous (n = 29)	*p*
Age, years, median (IQR)	69 (60–73)	68 (56–73)	0.39
Male sex, n (%)	31 (93.9)	11 (37.9)	<0.001
Pack-years, median (IQR)	30 (25–40)	20 (10–30)	0.010
Ever smokers, n (%)	33 (100)	22 (75.9)	0.003
Bilateral lesion, n (%)	19 (57.6)	8 (27.6)	0.022
Histopathological diagnosis (WHO 2024), n (%)			
Squamous cell carcinoma	33 (100)	0	—
Epithelium without dysplasia	0	18 (62.1)	—
Dysplasia	0	11 (37.9)	—
Clinical presentation, n (%)			
Mass lesion/leukoplakia/Reinke’s oedema/polyp	30 (90.9)/3 (9.1)/0/0	10 (34.5)/4 (13.8)/13 (44.8)/2 (6.9)	—
Carcinoma staging, n (%)			
T1/T2/T3/T4	14 (42.4)/0 (0)/11 (33.3)/8 (24.2)	—	—
G1/G2/G3	2 (6.1)/28 (84.8)/3 (9.1)	—	—
NBI vascular morphology, n (%)			
ELS perpendicular (high risk)	32 (97.0)	9 (31.0)	<0.001
Ni type 2/3/4/5	2 (6.1)/3 (9.1)/2 (6.1)/26 (78.8)	19 (65.5)/4 (13.8)/4 (13.8)/2 (6.9)	<0.001
Ni 4–5	28 (84.8)	6 (20.7)	<0.001
Ni 5	26 (78.8)	2 (6.9)	<0.001

IQR, interquartile range; ELS, European Laryngological Society. Continuous variables were compared with the Mann–Whitney U test, categorical variables with Fisher’s exact test (Ni types: χ^2^); dashes indicate comparisons defined in one group only. Dysplasia comprised 4 low-grade and 7 high-grade lesions; no papillomas occurred. Staging follows UICC/AJCC TNM 8th edition and WHO 2024; nodal status was not recorded prospectively.

**Table 2:** Per-gene accounting of all 62 specimens. No specimen failed for all four targets; RNA-quality failures therefore did not contribute to attrition, and all losses were transcript-specific non-detects (no amplification within 40 cycles).

Gene	Carcinoma: Evaluable	Carcinoma: Non-Detect	Non-Cancerous: Evaluable	Non-Cancerous: Non-Detect
*IL6*	32	1	25	4
*CXCL9*	33	0	23	6
*LIF*	30	3	25	4
*LIFR*	29	4	26	3

**Table 3:** Relative transcript abundance (2^−ΔΔCt^), median and number of evaluable specimens. High-risk patterns are ELS-perpendicular and the higher Ni categories of each contrast. Comparisons used the Mann–Whitney U test; the nominal significance level was 0.05, and q-values are Benjamini–Hochberg false discovery rates across all 20 estimable tests in this table; no comparison remained significant after correction (smallest q = 0.27). The ELS contrast within the carcinoma group is not shown because only one carcinoma had a longitudinal pattern and the comparison was not estimable; that specimen is discussed in Section 4.1.

Group	Contrast	Gene	High-Risk Pattern, Median (n)	Low-Risk Pattern, Median (n)	*p*	q
Carcinoma	Ni 4–5 vs. 1–3	*IL6*	5.97 (28)	5.20 (4)	0.805	0.894
		*CXCL9*	7.11 (28)	1.77 (5)	0.478	0.675
		*LIF*	1.77 (25)	0.21 (5)	0.169	0.437
		*LIFR*	0.19 (24)	0.45 (5)	0.414	0.675
	Ni 5 vs. 1–4	*IL6*	5.97 (26)	5.20 (6)	0.724	0.852
		*CXCL9*	5.63 (26)	16.92 (7)	0.949	0.949
		*LIF*	1.73 (24)	0.52 (6)	0.273	0.546
		*LIFR*	0.19 (22)	0.33 (7)	0.901	0.948
Non-cancerous	ELS perpendicular vs. longitudinal	*IL6*	10.79 (7)	0.18 (18)	0.025	0.271
		*CXCL9*	17.55 (9)	2.07 (14)	0.041	0.271
		*LIF*	15.37 (8)	2.04 (17)	0.175	0.437
		*LIFR*	2.61 (8)	0.49 (18)	0.261	0.546
	Ni 4–5 vs. 1–3	*IL6*	20.31 (5)	0.21 (20)	0.029	0.271
		*CXCL9*	17.37 (6)	3.99 (17)	0.319	0.580
		*LIF*	29.89 (6)	2.04 (19)	0.080	0.331
		*LIFR*	15.47 (6)	0.49 (20)	0.083	0.331
	Ni 5 vs. 1–4	*IL6*	52.85 (2)	0.23 (23)	0.107	0.356
		*CXCL9*	17.37 (2)	4.06 (21)	0.506	0.675
		*LIF*	14.58 (2)	2.15 (23)	0.600	0.750
		*LIFR*	13.50 (2)	0.60 (24)	0.443	0.675

**Table 4:** Diagnostic performance of NBI classifications and *IL6* expression for discriminating carcinoma from non-cancerous lesions (carcinoma as the positive class). PPV/NPV: positive/negative predictive value.

Classifier	Sensitivity, % (95% CI)	Specificity, % (95% CI)	PPV, % (95% CI)	NPV, % (95% CI)	Accuracy, % (95% CI)	Youden
ELS perpendicular	96.9 (84.3–99.4)	72.0 (52.4–85.7)	81.6 (66.6–90.8)	94.7 (75.4–99.1)	86.0 (74.7–92.7)	0.69
Ni 4–5 vs. 1–3	87.5 (71.9–95.0)	80.0 (60.9–91.1)	84.8 (69.1–93.3)	83.3 (64.1–93.3)	84.2 (72.6–91.5)	0.68
Ni 5 vs. 1–4	81.2 (64.7–91.1)	92.0 (75.0–97.8)	92.9 (77.4–98.0)	79.3 (61.6–90.2)	86.0 (74.7–92.7)	0.73
IL6 ≥ 0.52	93.8 (79.9–98.3)	56.0 (37.1–73.3)	73.2 (58.1–84.3)	87.5 (64.0–96.5)	77.2 (64.8–86.2)	0.50

All classifiers were computed on the subset evaluable for *IL6* (n = 57; 32 carcinoma, 25 non-cancerous); denominators are therefore common across rows. Confidence intervals are Wilson intervals. PPV and NPV are conditioned on the carcinoma prevalence of this surgical series and are not transportable to a screening setting. The *IL6* cut-off was derived and evaluated in the same sample, so its sensitivity and specificity are apparent values.

**Table 5:** Association of smoking exposure with transcript abundance, stratified by histological group.

Group	Gene	Pack-Years vs. Expression: ρ (*p*); n	Never-Smoker Median (n)	Ever-Smoker Median (n)	Ever vs. Never *p*
Cancer	*IL6*	0.29 (0.11); 32	-	-	-
	*CXCL9*	0.10 (0.59); 33	-	-	-
	*LIF*	0.29 (0.13); 30	-	-	-
	*LIFR*	0.06 (0.74); 29	-	-	-
Non-cancer	*IL6*	0.04 (0.84); 25	38.41 (6)	0.23 (19)	0.60
	*CXCL9*	−0.13 (0.56); 23	10.11 (7)	4.73 (16)	0.62
	*LIF*	−0.56 (**0.003**); 25	9.65 (7)	0.55 (18)	**0.003**
	*LIFR*	−0.33 (0.10); 26	3.26 (7)	0.46 (19)	0.17

Spearman ρ relates pack-years (a continuous measure of cumulative exposure) to 2^−ΔΔCt^ expression; n = evaluable pairs. The ever- versus never-smoker comparison (Mann–Whitney U) is shown for the non-cancer group only, because all carcinomas occurred in current or former smokers.

## Data Availability

De-identified data supporting the findings of this study (histological diagnosis, NBI category under both classification systems, sex, pack-years, T category and 2^−ΔΔCt^ values for all four transcripts) are provided as Supplementary Table S1. A completed STROBE checklist is provided as Supplementary File S1.

## Supplementary Materials

The following supporting information can be downloaded at: https://www.mdpi.com/article/10.3390/cancers18213545/s1, Table S1: De-identified analysis dataset for all 62 glottic lesions: histopathological diagnosis (WHO 2022), clinical and demographic covariates, NBI classification under both systems, and 2^−ΔΔCt^ values for IL6, CXCL9, LIF and LIFR with per-transcript evaluability status.; File S1: Completed STROBE checklist for cross-sectional studies, indicating where each of the 22 checklist items is reported in the manuscript.
