# Supplementary material for: Transcript Abundance of IL6, CXCL9, LIF and LIFR in Glottic Lesions: Relationship to Histology and to Narrow-Band Imaging Vascular Patterns
Source: Cancers (Basel). 2026 Nov 4;18(21):3545. doi: 10.3390/cancers18213545 (PMC13762002; doi:10.3390/cancers18213545)
Supplement: Supplementary file 1 [file cancers-18-03545-s001.zip › File S1.pdf]

Supplementary File S1 - Completed STROBE checklist for cross-sectional studies, indicating where each of the 22 checklist items is reported in the manuscript.

SECTION/TOPIC	ITEM NO	RECOMMENDATION	REPORTED IN MANUSCRIPT
**TITLE AND ABSTRACT**	1	(a) Indicate the study's design with a commonly used term in the title or the abstract	Abstract ("prospective, single-centre exploratory cohort")
		(b) Provide in the abstract an informative and balanced summary of what was done and what was found	Abstract
**INTRODUCTION**			
**BACKGROUND/RATIONALE**	2	Explain the scientific background and rationale for the investigation being reported	Section 1, paragraphs 1–4
**OBJECTIVES**	3	State specific objectives, including any prespecified hypotheses	Section 1, final paragraph (primary and secondary exploratory aims); Abstract
**METHODS**			
**STUDY DESIGN**	4	Present key elements of study design early in the paper	Section 2.1
**SETTING**	5	Describe the setting, locations, and relevant dates, including periods of recruitment, exposure, follow-up, and data collection	Section 2.1 (single tertiary centre; recruitment November 2020 – June 2023); Sections 2.2–2.3 (endoscopy on the day of surgery, intraoperative sampling)
**PARTICIPANTS**	6	(a) Give the eligibility criteria, and the sources and methods of selection of participants	Section 2.1 (inclusion and exclusion criteria; requirement for a fresh fragment; overlap with reference [21])
**VARIABLES**	7	Clearly define all outcomes, exposures, predictors, potential confounders, and effect modifiers. Give diagnostic criteria, if applicable	Section 2.2 (ELS and Ni classifications); Section 2.3 (histopathology, WHO 5th edition); Section 2.4 (transcript quantification); Section 2.5 (sex and smoking as potential confounders)
**DATA SOURCES/MEASUREMENT**	8*	For each variable of interest, give sources of data and details of methods of assessment (measurement). Describe comparability of assessment methods if there is more than one group	Sections 2.2–2.4; identical methods applied to both histological groups
**BIAS**	9	Describe any efforts to address potential sources of bias	Section 2.2 (blinded consensus scoring); Section 2.4 (non-detects not treated as missing data; calibrator set independent of the study lesions); Section 2.5 (sensitivity analyses, ever-smoker analysis); Section 4.3
**STUDY SIZE**	10	Explain how the study size was arrived at	Section 2.1 (no a-priori calculation; minimum detectable standardised difference 0.73 SD)
**QUANTITATIVE VARIABLES**	11	Explain how quantitative variables were handled in the analyses. If applicable, describe which groupings were chosen and why	Section 2.2 (Ni grouping 1–3 vs 4–5 and 5 vs 1–4); Section 2.4 ($2^{-\Delta\Delta Ct}$); Section 2.5 (log10 transformation in logistic models)
**STATISTICAL METHODS**	12	(a) Describe all statistical methods, including those used to control for confounding	Section 2.5
		(b) Describe any methods used to examine subgroups and interactions	Section 2.5 (histology-stratified comparisons with Benjamini–Hochberg correction); Section 3.3 (dysplasia-stratified analysis)
		(c) Explain how missing data were addressed	Sections 2.4 and 2.5 (non-detects; sensitivity analysis at the limit of detection); Table 2
		(d) If applicable, describe analytical methods taking account of sampling strategy	Not applicable (single-centre surgical series without complex sampling)
		(e) Describe any sensitivity analyses	Section 2.5; Section 3.2
**RESULTS**			

**PARTICIPANTS**	13*	(a) Report numbers of individuals at each stage of study—eg numbers potentially eligible, examined for eligibility, confirmed eligible, included in the study, completing follow-up, and analysed	Section 3.1; Table 2 (evaluable specimens per transcript)
		(b) Give reasons for non-participation at each stage	Section 2.1 (fresh fragment not obtainable without compromising the diagnostic specimen); Table 2 (transcript-specific non-detects)
		(c) Consider use of a flow diagram	Not provided; specimen accounting given in Table 2
**DESCRIPTIVE DATA**	14*	(a) Give characteristics of study participants (eg demographic, clinical, social) and information on exposures and potential confounders	Table 1
		(b) Indicate number of participants with missing data for each variable of interest	Table 2; Tables 3 and 5 (n per analysis); Table 1 footnote (nodal status not recorded)
**OUTCOME DATA**	15*	Report numbers of outcome events or summary measures	Section 3.1; Table 1
**MAIN RESULTS**	16	(a) Give unadjusted estimates and, if applicable, confounder-adjusted estimates and their precision (eg, 95% confidence interval). Make clear which confounders were adjusted for and why they were included	Section 3.2 (crude OR 2.06 and sex-adjusted OR 1.78 with 95% CI; rationale in Section 2.5)
		(b) Report category boundaries when continuous variables were categorized	Section 3.4 and Table 4 (IL6 cut-off ≥ 0.52)
		(c) If relevant, consider translating estimates of relative risk into absolute risk for a meaningful time period	Not applicable (cross-sectional design); predictive values in Table 4
**OTHER ANALYSES**	17	Report other analyses done—eg analyses of subgroups and interactions, and sensitivity analyses	Section 3.2 (limit-of-detection and ever-smoker analyses); Section 3.3 (stratified and dysplasia analyses); Section 3.4 (bootstrap ΔAUC, optimism correction); Sections 3.5–3.6
**DISCUSSION**			
**KEY RESULTS**	18	Summarise key results with reference to study objectives	Section 4, first paragraph; Section 4.1
**LIMITATIONS**	19	Discuss limitations of the study, taking into account sources of potential bias or imprecision. Discuss both direction and magnitude of any potential bias	Section 4.3; Section 4.2 (direction of bias from GAPDH normalisation)
**INTERPRETATION**	20	Give a cautious overall interpretation of results considering objectives, limitations, multiplicity of analyses, results from similar studies, and other relevant evidence	Sections 4.1–4.2; Section 5
**GENERALISABILITY**	21	Discuss the generalisability (external validity) of the study results	Section 4.3 (single-centre surgical series; predictive values not transportable; laboratory-specific cut-off)
**OTHER INFORMATION**			
**FUNDING**	22	Give the source of funding and the role of the funders for the present study and, if applicable, for the original study on which the present article is based	Funding statement; Conflicts of Interest statement
